# Increased Retention of Cardiac Cells to a Glass Substrate through Streptavidin–Biotin Affinity

**DOI:** 10.1021/acsomega.1c02003

**Published:** 2021-07-01

**Authors:** Kara A. Davis, Jensen Z. Goh, Andrea H. Sebastian, Brooke M. Ahern, Christine A. Trinkle, Jonathan Satin, Ahmed Abdel-Latif, Brad J. Berron

**Affiliations:** † Department of Chemical and Materials Engineering, University of Kentucky, Lexington, Kentucky 40506, United States; ‡ Department of Physiology, University of Kentucky, Lexington, Kentucky 40536, United States; § Department of Mechanical Engineering, University of Kentucky, Lexington, Kentucky 40506, United States; ∥ Gill Heart and Vascular Institute and Division of Cardiovascular Medicine, University of Kentucky and the Lexington VA Medical Center, Lexington, Kentucky 40506, United States

## Abstract

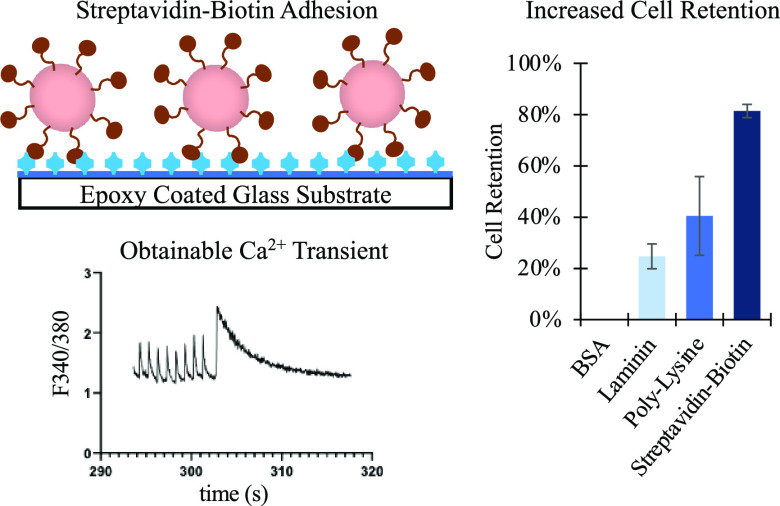

In vitro analysis of primary isolated adult cardiomyocyte physiological processes often involves optical imaging of dye-loaded cells on a glass substrate. However, when exposed to rapid solution changes, primary cardiomyocytes often move to compromise quantitative measures. Improved immobilization of cells to glass would permit higher throughput assays. Here, we engineer the peripheral membrane of cardiomyocytes with biotin to anchor cardiomyocytes to borosilicate glass coverslips functionalized with streptavidin. We use a rat cardiac myoblast cell line to determine general relationships between processing conditions, ligand density on the cell and the glass substrate, cellular function, and cell retention under shear flow. Use of the streptavidin–biotin system allows for more than 80% retention of cardiac myoblasts under conventional rinsing procedures, while unmodified cells are largely rinsed away. The adhesion system enables the in-field retention of cardiac cells during rapid fluid changes using traditional pipetting or a modern microfluidic system at a flow rate of 160 mL/min. Under fluid flow, the surface-engineered primary adult cardiomyocytes are retained in the field of view of the microscope, while unmodified cells are rinsed away. Importantly, the engineered cardiomyocytes are functional following adhesion to the glass substrate, where contractions are readily observed. When applying this adhesion system to cardiomyocyte functional analysis, we measure calcium release transients by caffeine induction at an 80% success rate compared to 20% without surface engineering.

## Introduction

In a critical effort to displace heart disease from its perennial position atop the leading causes of death in the developed world,^1^ cardiac research has turned its focus to cellular function. The syncytial nature of the heart allows the use of isolated cardiomyocytes as surrogates for heart chamber function and for the interrogation of cellular and molecular mechanisms. For example, cytosolic Ca^2+^ is a central determinant of heart function, and Ca^2+^ dyshomeostasis is associated with reduced contraction and arrythmias.^2^ Therefore, methods to inhibit and regulate the currents through the L-type calcium channels in cardiomyocytes (CMs) are of great interest.^2−5^ Multiple, interdependent processes regulate the sarcoplasmic reticulum Ca^2+^ load. Hence, precise measurement of the Ca^2+^ load serves as a key integrative surrogate measure for heart function and prediction of disease. A standard bioassay of sarcoplasmic reticulum Ca^2+^ load requires CM immobilization onto an optically clear glass surface.

CMs offer an added level of complexity compared to other primary cells due to their unique elongated shape and rigidity.^6^ CMs are prone to damage when exposed to changes in temperature,^7^ prolonged studies, and routine separation and mixing techniques. As a result, CMs require long separations by gravity or very gentle centrifugation,^7^ while many other primary cells are readily pelleted with minimal impact on cellular function.^8^ In addition to the unique sensitivity of CMs, working times are limited as basic CM properties such as morphology^7^ and electrical and contractile forces are lost in culture.^9^ These short CM working times do not facilitate natural cell–substrate adhesion formed over several hours in a culture common to other cell types. While many labs have adapted the use of “cellular glues,” Dvornikov *et al*. noted that while performing cellular stretching, any stretch beyond 20% resulted in cell detachment despite the use of MyoTak cell glue.^10^ In this work, we develop a method where CMs remain immobilized despite the rapid exchange of stimulating solutions.

**Figure 1:**
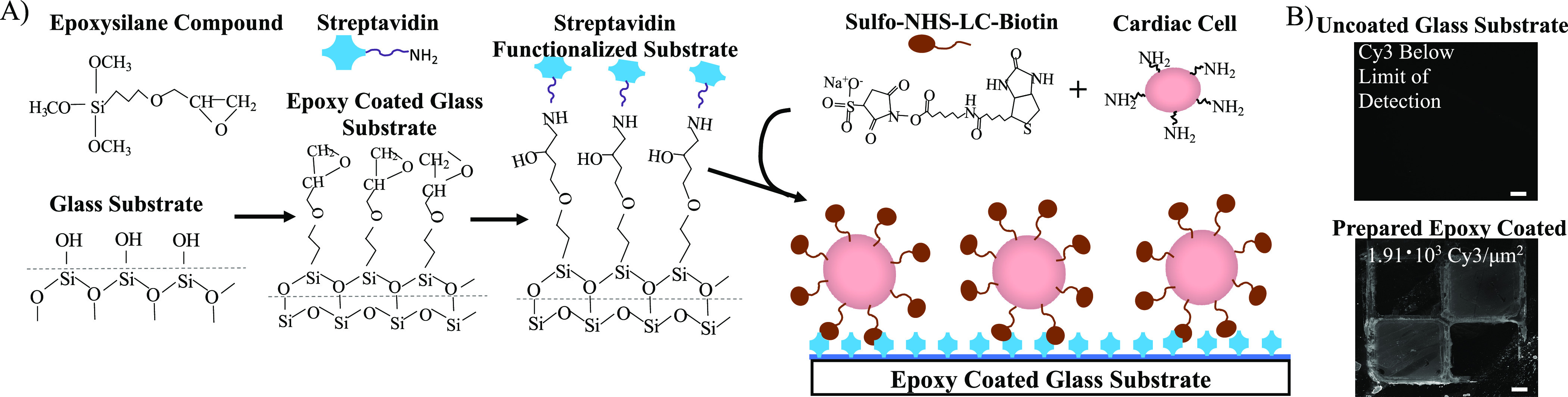
Functionalization of a glass substrate to increase cell retention. (A) Epoxy coating of a glass substrate followed by streptavidin conjugation and binding of biotinylated cells. (B) Fluorescent imagining of SA-Cy3 coating on epoxy slides and negative control/unmodified slides. Streptavidin density determined using the Cy3 calibration slide (scale bar = 1 mm).

Cell surface engineering has emerged across many biological applications as a way to adhere cells to targeted areas. Specifically, it has been used to adhere or target cells to sites of inflammation,^11−13^ the myocardium,^14−16^ and various substrates.^17−19^ One of the most common interactions used in cell surface engineering is that of between biotin and streptavidin^18,20−23^ due to their high affinity and ability to form a single streptavidin bridge between up to four biotinylated molecules. In particular, Iwasaki and Ota showed that microarrays of functional, adhered cells could be achieved by patterning streptavidin on a silicon-based surface followed by incubation with biotinylated cells.^18^ In this work, we applied this simple concept by contacting biotinylated cardiac cells to streptavidin-functionalized surfaces, and we observed a significant increase in cell immobilization despite high fluidic flow rates (Figure 1).

Specifically, we studied the adhesive force of biotin–streptavidin when applied to a cell-to-glass adhesion system. To accelerate the development of the adhesion system, we first developed the principles of this system using a relatively robust and easy to culture cardiomyoblast cell line (H9C2). We first determined the density of streptavidin bound to the glass substrate and the impact of biotin on cardiomyoblast viability. Then, we determined the percent retention of cells when subjected to increasing fluidic flow rates. We then applied the adhesion system to freshly isolated primary CMs, where the adhesion enables reliable in situ microscopic observation of CM functional assays. The health and function of the CMs were confirmed through morphological assessments and the shear-induced contraction of adhered CMs. Overall, the high affinity between biotin and streptavidin allowed for an adhesion force capable of immobilizing cells onto a glass substrate while exposed to fluidic flow rates up to 160 mL/min. Using a microfluidic device with the adhesion system, cells were retained in the field of view while the buffer in contact with the cells is exchanged in less than a second. Finally, we show that following the introduction of the effect of caffeine flow, CMs remain fully immobilized and a calcium transient can be obtained at a significantly higher rate.

## Results and Discussion

**Figure 2:**
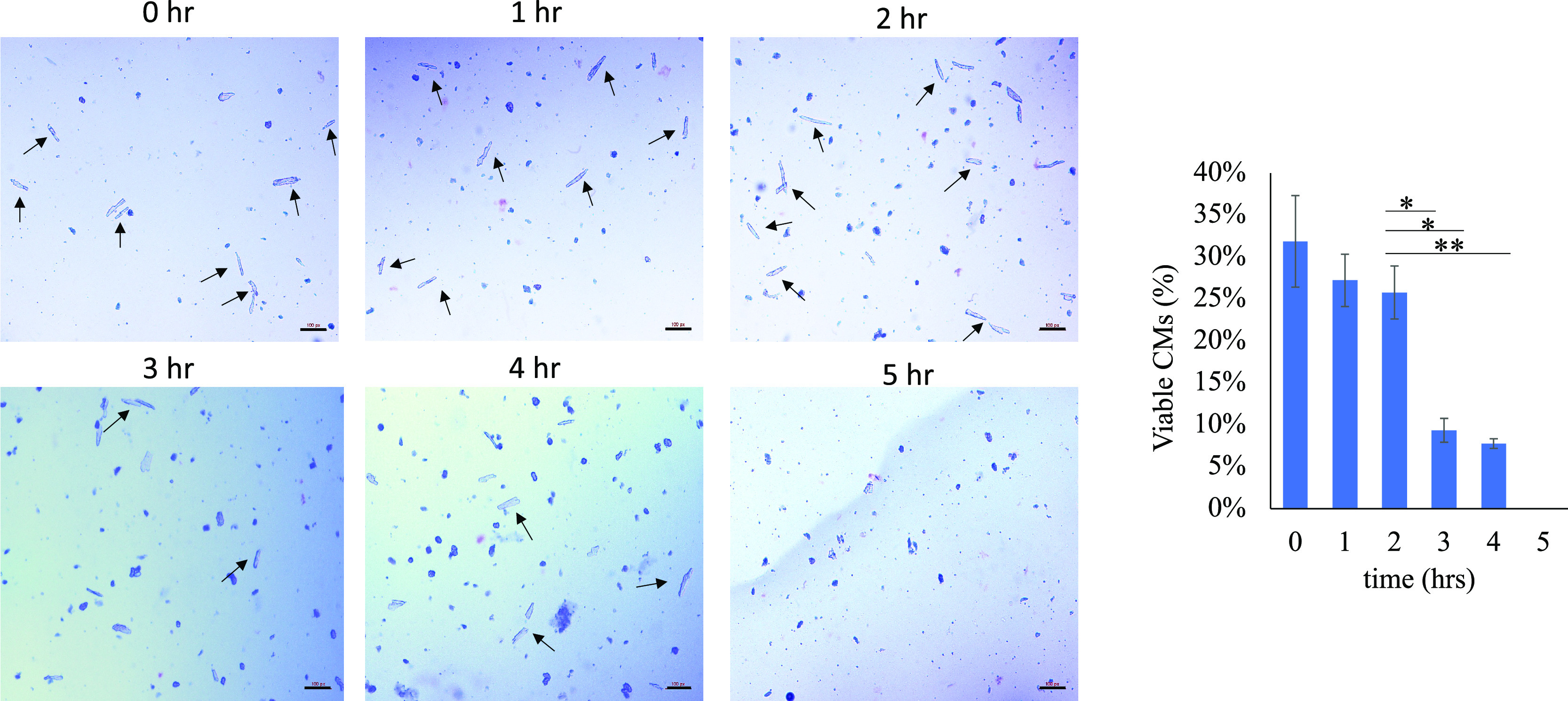
Viability of cardiomyocytes determined by Trypan Blue staining during the first 5 h following retrieval. Black arrows indicate CMs with an intact membrane. (p* < 0.05, p** < 0.01, and scale bar = 100 μm).

Our goal was to immobilize cardiac cells on glass microscope slides so that they would remain in a fixed location during the rapid exchange of liquid solutions. Rapid solution exchange is necessary in primary cardiomyocyte experiments when studying active contraction, but high fluid shear stresses generated during exchange steps normally cause cardiomyocytes to delaminate. Additionally, adult mammalian ventricular cardiomyocytes must be used within a narrow window of time because overnight or longer culture leads to a “de-differentiated” phenotype.^24^ In Figure 2, we emphasize the change in cell viability after just 3 h.

Due to their short working times, there is a significant need to perform studies rapidly with as many of these precious cells as possible. Any loss of cells from the experimental view due to shear requires additional experimental runs and lost functional time. By applying our adhesion system (Figure 1), this allows environmental changes in microscopy-enabled cell function analyses without losing sight of the target cell. A biotin/streptavidin system was chosen because the biotin and streptavidin linkage has one of the strongest known noncovalent biological affinities.^25^ By utilizing glycidyl silane monolayer chemistry, a glass surface can covalently bind free amine groups on streptavidin, and the resulting streptavidin-coated glass substrate can then strongly bind biotinylated cells (Figure 1).

In order to confirm that streptavidin was covalently bound to epoxy-functionalized glass surfaces, Cy3-conjugated streptavidin (SA-Cy3) was deposited onto freshly prepared epoxy. Using a Cy3 Full Moon Biosystems scanner calibration slide, the fluorescent signal obtained from an Affymetrix 428 Array scanner could be directly related to the fluorophore density on the glass surface. Concentration of SA-Cy3 was varied and it was determined that the optimum concentration of SA-Cy3 was 20 μg/mL (Figure S1). While unmodified glass surfaces had a fluorophore density below the level of detection, freshly prepared epoxy-coated slides modified with 20 μg/mL SA-Cy3 showed effective conjugation (Figure 1) with a fluorophore density of 1.91 × 10^3^ Cy3/μm^2^. This loading density of SA-Cy3 is well above the observed maximum SA binding of biotinylated H9C2 (114 ± 21 SA/μm^2^, Figure S2).

## eSA–Biotin Allows for Higher Immobilization Than Alternate Methods

**Figure 3:**
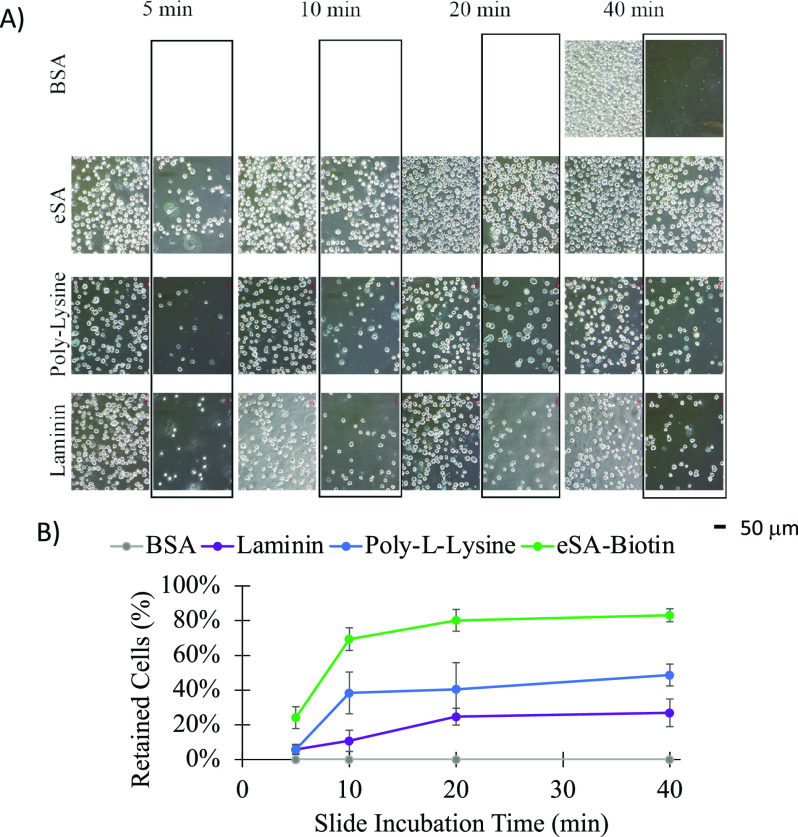
Retention of H9C2 following PBS rinsing when incubated with laminin and poly-L-lysine cover glass compared to biotinylated H9C2 on an eSA-functionalized cover. A BSA-blocked glass substrate was used as a control. (A) Microscopic images of cell retention at incubation times of 5, 10, 20, and 40 min (scale bar = 50 μm). (B) Summary plot of cell retention versus incubation time. Retention results are an average of three replicates with three areas counted per coverslip (approximately 150 cells per replicate).

Both laminin and poly-L-lysine-coated cover glass are commonly used for the adherence of unmodified cells in a culture.^7^ While these surfaces allow for increased cell retention over uncoated cover glass, retention is still limited. After pipetting 500 μL of PBS, the highest observed retention of unmodified H9C2 on BSA, laminin, or poly-lysine-coated cover glass was approximately 0, 30, and 50%, respectively (Figure 3). By contrast, cell retention above 80% was achieved for biotinylated H9C2 on eSA cover glass.

Retention of cells versus incubation time was tested next. H9C2 cells were incubated on each substrate at varying times (5, 10, 20, and 40 min). For the laminin group, statistically significant changes in retention were observed only when increasing incubation from 10 to 20 min (statistical analyses in the Supporting Information, Table S1). Based on Figure 3, cell retention with the eSA–biotin system improved significantly up to a 20 min incubation, while no statistical difference in cell retention was observed with longer incubations of cardiomyoblasts on the glass substrate. Overall, the eSA–biotin system achieved the highest retention rate at any given duration of incubation.

Based on the previous results using H9C2 cardiomyoblasts, we tested our engineered adhesion system on primary CMs using a 20 min incubation time. Given that primary CMs are functional for only a few hours in vitro (Figure 2), the short incubation time required to achieve adhesion is advantageous. Adult CMs were incubated with both BSA-blocked and eSA-functionalized cover glass. While CMs did not adhere to the BSA-blocked cover glass (Figure 4Video S1), high retention was observed for the eSA–biotin system following rapid pipetting of 500 μL of PBS, demonstrating that the use of biotin–streptavidin affinity is sufficient to immobilize cardiac cells onto a glass substrate under the flow conditions common to calcium studies on CMs.

**Figure 4:**
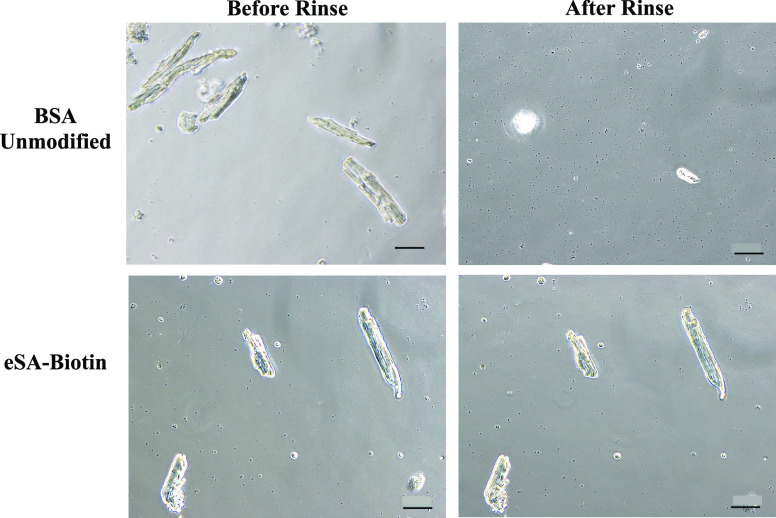
Retention of primary cardiomyocytes following the introduction of PBS for unmodified cells on a BSA-blocked substrate versus biotinylated cells on a eSA-functionalized substrate (scale bar = 50 μm).

## Cells Remain Immobilized Despite Rapid Change in Concentration

A variety of experimental protocols calls for rapid superfusion of a bolus of drugs, inhibitors, or release agents (such as caffeine). However, if the cell is not immobilized onto the surface or the shear from the superfusion is too strong, the cell is washed away from the viewing surface. The required perfusion rates can be higher than what would be experienced under normal medium change conditions; these rapid concentration changes lead to increased shear stress that attempt to peel cells from their adhered surfaces. To analyze this phenomenon, we assessed the rate in which concentration changes as it reaches the cell surface and its impact on cell retention.

**Figure 5:**
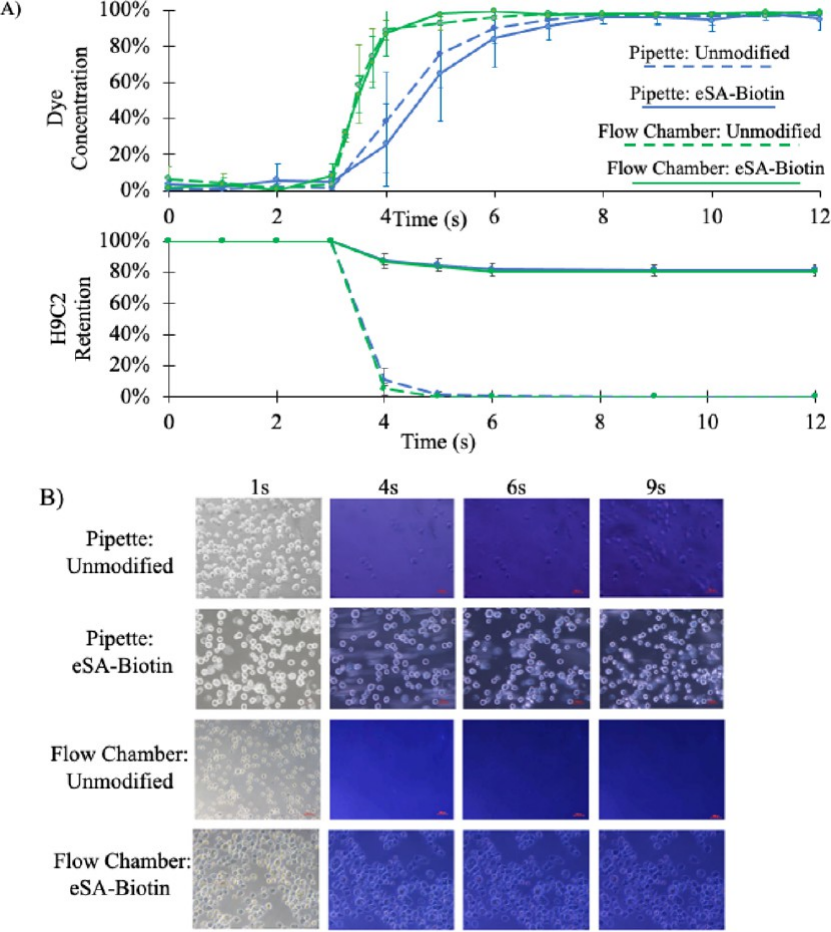
Time for dye to reach full concentration through injection with hand pipetting versus perfusion with a 160 mL/min parallel-plate flow chamber. (A) Retention of unmodified H9C2 on a plain glass substrate versus NHS-biotin-modified H9C2 on eSA. (B) Microscopic images of cell retention captured at 1, 4, 6, and 9 s following the introduction of trypan blue.

To see a clear change from the suspension buffer to the introduction of a concentrated solution, trypan blue dye was used as a model agent for its ease of optical measurement. H9C2 cells were incubated for 20 min with either unmodified or eSA-modified cover glass and then placed on a Nikon Ti-U inverted microscope as 0.05% trypan blue was introduced to the cell surface using a traditional fixed volume pipettor. Change in color was used to determine the concentration of trypan blue as a function of time (Figure 5). While the concentration change trend was the same for unmodified cells versus the eSA–biotin system, cell retention is only observed in the eSA–biotin case (Table S1).

Additionally, we wanted to show that H9C2 would remain adhered with our eSA–biotin system when subject to more rapid changes in concentration. Here, a parallel-plate flow chamber was used to control the rate at which solution was introduced to the cell surface. Specifically, 0.1% trypan blue was pumped across both an unmodified surface and the eSA–biotin system at a rate of 160 mL/min (Figure 5). As seen in Figure 5, the rate change of dye concentration was significantly increased (Table S1), while no statistical change in cell retention was observed. Indeed, Figure 5 shows that even under high fluidic shear (260 dynes/cm^2^), ∼82% of the initially deposited cells remain in the field of view.

## Biotinylation Does Not Impact Cell Viability and Function

**Figure 6:**
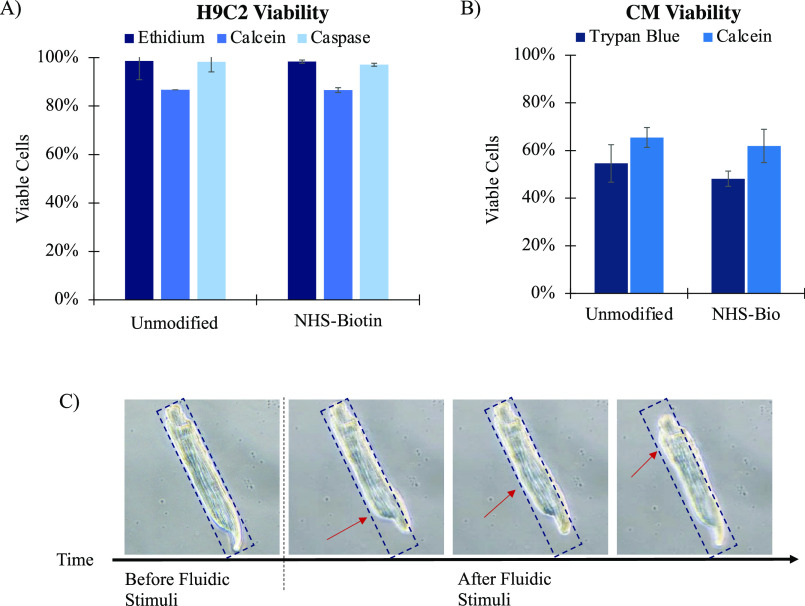
(A) Viability of unmodified H9C2 versus NHS-biotin-modified H9C2 through calcein, ethidium, and caspase assays. (B) Viability of unmodified CM versus NHS-biotin-modified CM determined by trypan blue (p* = 0.29) and calcein assay (p* = 0.60). (C) Contraction of adhered cardiomyocytes following fluidic stimuli. The arrow indicates the position of the deformation wave during cell contraction.

To immobilize cardiac cells onto the epoxy-streptavidin (eSA) glass substrate, cells were first biotinylated using NHS-biotin. NHS-biotin binds to the cells’ surface through a covalent linkage with free amine groups found on cell surface proteins (Figure 1). In order to evaluate the impact of NHS-biotin on the viability of H9C2, we performed a calcein assay for esterase activity, an ethidium assay for membrane integrity, and a caspase assay to evaluate apoptosis (Figure 6). A *t* test showed no significant difference between uncoated cells and NHS-biotin-modified H9C2 in terms of esterase activity, membrane integrity, and apoptosis activation.

Harsh processing for CM retrieval results in a lower overall viability than observed with the H9C2 cell line used in the studies from Figure 6A. However, the viability of CMs was not statistically impacted by NHS-biotin modification based on trypan blue/integrity and calcein/esterase assays (Figure 6B). Another concern is contractile function: healthy adult CMs spontaneously contract when exposed to fluidic shear.^26^ In our experiments, NHS-biotin-modified CMs remained quiescent until stimulated by the fluidic shear of PBS pipetted onto the surface (Figure 6C and Video S1). Following the introduction of shear with a stream of PBS, CMs spontaneously contracted. This observation supports the hypothesis that adult CMs may be modified with NHS-biotin and immobilized onto an eSA surface and retain contractile cell function.

## eSA–Biotin System Allows Higher Ca^2+^ Transient Observation Efficiency

**Figure 7:**
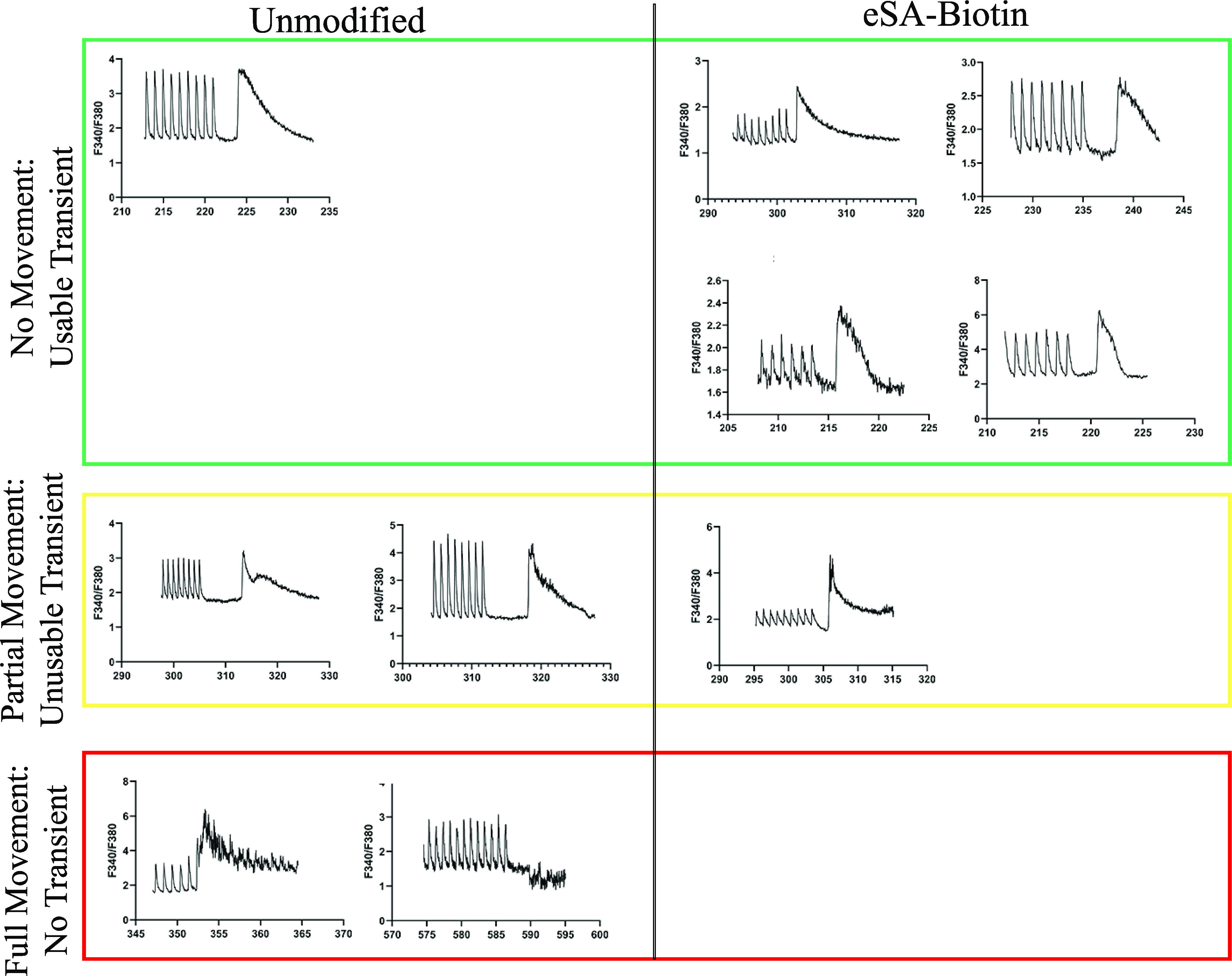
Ability to obtain Ca^2+^ transients following the introduction of a 10 mM caffeine puff. Systems are compared based on CM movement and observed Ca^2+^ transient. CM movement is directly related to if a transient is useable or not. (*n* = 5 per sample group; *y* axis = F340/F380, *x* axis = time(s)).

We challenged our adhesion system with a representative assay of CM health that compares the amount of calcium released because of an electrical signal to the total calcium present in the CM. The calcium released following electrical pulses was quantified, and then each CM was induced to release all its calcium stored in the sarcoplasmic reticulum through rapid addition of caffeine.^27,28^ In a typical experiment on unmodified CMs, only ∼20% of CMs were retained in the field of view for measurement (Figure 7). More commonly, there was partial movement of the CM, which induced artifacts in the calcium measurement, or the rapid addition of caffeine moved the CMs from the focal plane, which precluded quantitation. In contrast, when our eSA–biotin system was used to anchor the CMs to the slides, all the CMs were held in the field of view and 80% of CMs yielded artifact-free calcium transients.

The short functional lifetime of CMs following a lengthy retrieval process motivates the need for an efficient and rapid measurement of CM function. High failure rates due to the movement of unmodified CMs across the slide will reduce the number of CM function measurements per animal and reduce the statistical power of a given study. Based on the results in Figure 7, a CM calcium analysis will yield data four times as often when using eSA–biotin than an unmodified CM.

## Conclusions

By utilizing biotin-streptavidin affinity, we immobilize cells onto a surface without impacting their viability or functionality. Cells were retained at a rate of 80% in an analytical microfluidic device under flow rates of up to 160 mL/min and magnitudes of fluidic shear exceeding physiological conditions. When considering the study of CMs under high magnification, the field of view will typically only contain a single cell. This adhesion system allows the rapid (<1 s) exchange of an analyte solution while supporting the likelihood that the cell under observation will be retained. Overall, we demonstrated that this method can be applied directly to the measurement of calcium signaling in individual CMs and results in increased data collection efficiency from 20 to 80% by binding a biotinylated CM to a streptavidin surface. The overarching significance of these methods extends beyond studying primary CMs to the increasingly used induced pluripotent derived CMs in mechanistic and therapeutic studies.^29−31^

## Materials and Methods

### Glass Substrate Preparation

Both glass microscope slides (VWR) and cover glass (Fisher) were cleansed in ethanol for 30 min followed by sterilization by plasma etching. Following sterilization, an epoxy coating was applied to the glass substrates (adapted from Tsukruk *et al*.^32^). Briefly, glass substrates were incubated in an epoxysilane solution of 1% (3-glycidoxypropyl)trimethoxysilane (Sigma-Aldrich) in toluene (Sigma-Aldrich) for 24 h. Following incubation, glass was rinsed several times with toluene and ethanol then allowed to dry. After drying, slides were blocked for nonspecific binding with 5 mg/mL bovine serum albumin in PBS (BSA) for 40 min. In order to verify epoxy coating and the ability to bind SA, varying concentrations of streptavidin-Cy3 (SA-Cy3, Invitrogen) in PBS (0.1, 02, 1, 2, 10, 20, 100, and 200 μg/mL) were incubated for 40 min onto epoxy-coated slides using altering wells and then washed with PBS. Freshly prepared epoxy-coated glass was compared to uncoated microscope slides. Arrays were analyzed using an Affymetrix 428 Array scanner and Image J software. A Cy3 calibration slide (Full Moon Biosystems) was used to determine the average fluorophore density for each sample (Figure S1). An optimal concentration of 20 μg/mL SA-Cy3 was determined and used for all further studies.

To compare retention of cells on unmodified glass to retention on various functionalized glass substrates, cover glass was also coated in laminin and poly-L-lysine. Cover glass was sterilized by ethanol and allowed to dry. Then, cover glass was incubated with either 0.1 mg/mL poly-L-lysine (Sigma-Aldrich) in PBS for 20 min or 5 μg/mL laminin (Gibco) in PBS for 2 h both at 37 °C. Cover glass was rinsed with molecular-grade water and allowed to dry.

### H9C2 Biotinylating and Viability

Rat cardiac myoblasts (H9C2, ATCC CLR-1446) were cultured in Dulbecco’s modified Eagle’s medium (DMEM, VWR) supplemented with 10% fetal bovine serum (FBS, VWR) and 1% penicillin/streptomycin (VWR) at 37 °C and 5% CO_2_. Cells were seeded in T-182 cm^2^ tissue culture flasks (VWR) and grown to approximately 80% confluence prior to use. Cells were aspirated using 0.25% trypsin–EDTA 1X (VWR) and then resuspended in 5 mL of the medium to neutralize trypsin. Cells were pelleted at 400 × g and 4 °C for 3 min, washed three times in 1 mL of PBS, and then resuspended in PBS for experiments.

Biotin was nonspecifically, covalently bound to proteins on the surface of the cell using a biotin-succinimidyl ester conjugate (NHS-biotin; EZ-link Sulfo-NHS-LC-biotin, Thermo Fisher). NHS-biotin was used at a concentration of 1 mM in PBS for 40 min with a volume of 500 μL per million cells. Biotin conjugation was performed at room temperature for primary CMs or on ice for H9C2. Cells were then rinsed and resuspended with PBS or complete Tyrode’s solution for H9C2 or primary CMs, respectively.

In order to verify the ability to coat biotinylated cells with SA, biotinylated cells were incubated with SA-PE at a concentration of 1–50 μL of PBS for 40 min. Samples of 10,000 events were analyzed by flow cytometry, and the PE mean fluorescence of SA-PE-tagged samples was recorded. BD QuantiBRITE PE quantitation beads were then used to quantify PE fluorescence by vortexing in 500 μL of PBS and analyzing the PE signal. The linear relationship between the PE signal and PE florescence was determined using QuanitBRITE PE (Figure S2) counting beads. Per Invitrogen, SA-PE is conjugated at a 1:1 ratio of SA to PE. Using the ratio and mesenchymal stem cell (MSC) diameter recorded after cell culture, SA density on the MSC surface was determined.

Following biotin conjugation, viability of H9C2 was tested using three different methods: calcein AM, ethidium homodimer-1 (Eth-1), and CellEvent Caspase-3/7. Unmodified H9C2, used as a control population and biotinylated H9C2 were separated into samples of approximately 25,000 cells and incubated at room temperature with each of the viability assays: calcein AM at 1 μL in 10 mL of PBS for 30 min, Eth-1 at 2 μL in 1 mL of PBS for 10 min, and caspase at 1 μL in 1 mL of PBS for 25 min. Cells were then rinsed, resuspended in PBS, and imaged using a Nikon Ti-U inverted microscope. Unmodified cardiomyocyte viability was also observed across 5 h using trypan blue at 100 μL in 100 μL cell suspension and then imaged immediately.

### Ventricular Myocyte Isolation and Biotinylating

Adult ventricular cardiomyocyte isolation was performed as in a previous study.^27^ Prior to heart excision, mice were anesthetized with ketamine+xylazine (90 + 10 mg/kg, intraperitoneally). Hearts were excised and immediately perfused on a Langendorff apparatus with a high-potassium Tyrode buffer and then digested with 5 to 7 mg of liberase (Roche). After digestion, atria were removed and left ventricular myocytes were mechanically dispersed in a stop buffer to quench the enzymatic reaction (high K basal perfusion buffer, FBS; 10 mM CaCl2). Calcium concentrations were gradually restored to physiological levels in a stepwise fashion (10 mM CaCl2 to 100 mM CaCl2), and only quiescent ventricular myocytes with visible striations and the absence of membrane blebs were used for adhesion studies. A vial containing a suspension of CMs was placed at approximately 45° and allowed to separate by gravity for 40 min. The supernatant was removed by pipetting, and cells were resuspended in 1 mM NHS-biotin, gently pipetted and allowed to incubate for 40 min. CMs were again placed at approximately 45° and allowed to separate by gravity. CMs were resuspended for further analysis.

Unmodified CMs, used as a control population, and biotinylated CMs were incubated at room temperature with each viability assay: calcein AM at 1 μL in 10 mL of PBS for 30 min and trypan blue at 100 μL in 100 μL of cell suspension imaged immediately. Unmodified CM viability was also observed across 5 h using trypan blue as described above.

All experiments were approved by the University of Kentucky IACUC in accordance with the NIH Guide for the Care and Use of Laboratory Animals (DHHS publication No. [NIH] 85-23, rev. 1996).

### Cell Incubation with Cover Glass

Biotinylated H9C2 cells were allowed to incubate with eSA cover glass for varying amounts of time (5, 10, 20, and 40 min). For comparison, unmodified H9C2 were incubated with laminin or poly-L-lysine-coated cover glass for the same time. Unmodified H9C2 incubated with BSA-blocked cover glass was used as a control. Following incubation time, cells were subject to fluidic shear by dispensing 500 μL of PBS in ∼1 s using an Eppendorf fixed volume pipettor. Images before and after PBS rinsing were captured with a Nikon Ti-U inverted microscope and used to quantify retention of cells. Unless otherwise stated, all studies used a 20 min incubation time for cells on modified cover glass. The study was repeated using unmodified CM on BSA-blocked cover glass versus biotinylated CMs on eSA cover glass.

### Rapid Change in Cell Surface Concentration

Biotinylated H9C2 were incubated on BSA-blocked and eSA microscope slides for 20 min. Unmodified cells on BSA-blocked microscope slides were used as a control. Following incubation, 500 μL of a blue dye solution, 0.05% trypan blue (Sigma-Aldrich) in PBS, was introduced to cells using an Eppendorf fixed volume pipettor. TinyTake software was used to record video from a Nikon Ti-U inverted microscope as the change in dye concentration reached the cell surface. A relative concentration of the dye was then determined by analyzing images at every second and determining the relative intensity of pixels using Image J. The pixel level was then normalized for each sample and a percent change from the initial to the final dye level was determined.

We further analyzed the CM adhesion in a flow chamber model. A GlycoTech Model 31-010 parallel-plate flow chamber, with a 0.005 inch gasket thickness, was used to increase the speed at which dye is introduced to the cell surface. Due to the configuration of the microfluidic device, the blue dye concentration was increased to 0.1% trypan blue in PBS to allow for optimal visualization of the change in dye concentration. The microfluidic device was operated at a maximum flow rate of 160 mL/min.

### Calcium Pathway Change Measurement

Both biotinylated and unmodified wild-type mouse ventricular CMs were used to obtain calcium transients. For both cases, cells were loaded with cell permeable fura2-AM (Invitrogen) at 1.0 Hz to determine transient amplitude, upstroke velocity, and the rate of decay. All measurements were made following more than 2 min of conditioning of 1 Hz field stimuli to induce a steady state. Transients were recorded at 1 Hz. For caffeine-induced transients, 10 mM caffeine in Tryode’s solution was introduced to the cell surface. All Ca^2+^ transient data were analyzed using IonOptix IonWizard 6.3. Background fluorescence for 380 and 340 nm wavelengths were determined from cell-free regions. Data are expressed as F340/380 and were corrected for background noise.

### Statistical Approach

Statistical analysis for all studies was performed using *t* test analysis where a *p* value of less than 0.05 was considered significant. All studies, other than Figure 3, used *n* = 5 samples. In Figure 3, 3 images were collected for each sample group (approximately 150 cells). The experiment was then repeated a total of three times.

## Supporting Information Available

(Supplementary Video S1) Cardiomyocytes remaining immobilized despite the rapid change in concentration: in the eSA–biotin model, cardiomyocytes can be seen contracting in response to fluidic shear; this confirms that cell function is maintained (MP4)
Tables listing *p* values for statistical analysis performed for Figures 1, 3, 5, and 6; statistical analysis was performed using *t* test and *n* = 5 samples for all study groups; (Figure S1) Cy3 calibration curves used to determine the density of Cy3 on an epoxy-coated glass substrate; (Figure S2) flow cytometry data for BD QuantiBRITE PE used to determine the maximum SA density on a H9C2 cell; (Figure S3) enlarged cell images from Figure 3; (Figures S4 and S5) representative microscopic images used to determine viability in Figure 6 (PDF)
