# Supplementary material for: Increased Retention of Cardiac Cells to a Glass Substrate through Streptavidin–Biotin Affinity
Source: ACS Omega. 2021 Jul 1;6(27):17523–30. doi: 10.1021/acsomega.1c02003 (PMC8280672; doi:10.1021/acsomega.1c02003)
Supplement: Supplementary file 2 — ao1c02003_si_002.pdf [file ao1c02003_si_002.pdf]

Increased Retention of Cardiac Cells to Glass Substrate through Streptavidin-Biotin Affinity

Kara A. Davis[1], Jensen Z. Goh[2], Andrea H. Sebastian[2], Brooke M. Ahern[2], Christine A. Trinkle[3], Jonathan Satin[2], Ahmed Abdel-Latif[4] and Brad J. Berron*[1]

[1]Department of Chemical and Materials Engineering, University of Kentucky, Lexington, Kentucky, USA

[2]Department of Physiology, University of Kentucky, Lexington KY, USA

[3]Department of Mechanical Engineering, University of Kentucky, Lexington KY, USA

[4]Gill Heart and Vascular Institute and Division of Cardiovascular Medicine, University of Kentucky and the Lexington VA Medical Center, Lexington, KY, USA

Correspondence to:

Brad J. Berron, email: brad.berron@uky.edu

Table S1. Statistical comparison using Student t-test for Figures 1, 3 and 6 .

Figure	Sample 1	Sample 2	Argument	P - value
1	Unmodified Glass	Epoxy Coated Glass	Cy3/μm^2	
2	Untouched H9C2	Biotinylated H9C2	Viability: Calcein<br>Viability: Ethidium<br>Viability: Caspase	0.6965<br>0.1414<br>0.6395
3	Untouched Cardiomyocyte	Biotinylated Cardiomyocyte	Viability: Trypan Blue	0.2942
			Viability: Calcein	0.6016
4	PBSA Untouched H9C2	eSA-Biotin System	Retention: 5 min<br>Retention: 10 min<br>Retention: 20 min<br>Retention: 40 min	**0.0045**<br>**0.0062**<br>**0.0028**<br>**0.0004**
	Laminin Untouched H9C2	eSA-Biotin System	Retention: 5 min<br>Retention: 10 min<br>Retention: 20 min<br>Retention: 40 min	**0.0011**<br>**0.0019**<br>**0.0008**<br>**0.0031**
	Poly-L-Lysine Untouched H9C2	eSA-Biotin System	Retention: 5 min<br>Retention: 10 min<br>Retention: 20 min<br>Retention: 40 min	**0.0010**<br>**0.0337**<br>**0.0405**<br>**0.0045**
	PBSA @ t	PBSA @ t+Δt	Retention: 5 to 10 min<br>Retention: 10 to 20 min<br>Retention: 5 to 10 min	N/A*<br>N/A*<br>N/A*
	Laminin @ t	Laminin @ t+Δt	Retention: 5 to 10 min<br>Retention: 10 to 20 min<br>Retention: 5 to 10 min	0.2977<br>**0.0394**<br>0.7049
	Poly-L-Lysine @ t	Poly-L-Lysine @ t+Δt	Retention: 5 to 10 min<br>Retention: 10 to 20 min<br>Retention: 5 to 10 min	**0.0380**<br>0.8637<br>0.4604
	eSA-Biotin @ t	eSA-Biotin @ t+Δt	Retention: 5 to 10 min<br>Retention: 10 to 20 min<br>Retention: 5 to 10 min	**0.0026**<br>**0.0480**<br>0.9075

Red Text: P*<0.05

Red Text and Background: P**<0.01

N/A*: No change. P-value cannot be calculated

Table S2. Statistical comparison using Student t-test for Figure 5.

Figure	Sample 1	Sample 2	Argument	P -value
5	Pipette: Unmodified	Pipette: eSA-Biotin System	Dye Concentration: 3 s	0.2303
			Dye Concentration: 4 s	0.4608
			Dye Concentration: 5 s	0.4829
			Dye Concentration: 6 s	0.5080
			Dye Concentration: 7 s	0.3899
			Dye Concentration: 8 s	0.7165
	Microfluidic Device: Unmodified	Microfluidic Device: eSA-Biotin System	Dye Concentration: 3 s	0.3586
			Dye Concentration: 4 s	0.8348
			Dye Concentration: 5 s	0.1235
			Dye Concentration: 6 s	0.0986
			Dye Concentration: 7 s	0.5230
			Dye Concentration: 8 s	0.3527
	Pipette: eSA-Biotin System	Microfluidic Device: eSA-Biotin System	Dye Concentration: 3 s	0.5569
			Dye Concentration: 4 s	**0.0026**
			Dye Concentration: 5 s	**0.0496**
			Dye Concentration: 6 s	0.1020
			Dye Concentration: 7 s	0.1570
			Dye Concentration: 8 s	0.2841
	Pipette: Unmodified	Pipette: eSA-Biotin System	Retention: 3 s	N/A*
			Retention: 4 s	**0.0000**
			Retention: 5 s	**0.0003**
			Retention: 6 s	**0.0007**
	Microfluidic Device: Unmodified	Microfluidic Device: eSA-Biotin System	Retention: 3 s	N/A*
			Retention: 4 s	**0.0000**
			Retention: 5 s	**0.0000**
			Retention: 6 s	**0.0000**
	Pipette: eSA-Biotin System	Microfluidic Device: eSA-Biotin System	Retention: 3 s	N/A*
			Retention: 4 s	0.8411
			Retention: 5 s	0.6019
			Retention: 6 s	0.5924

Red Text: P*<0.05

Red Text and Background: P*<0.01

N/A*: No change. P-value cannot be calculated

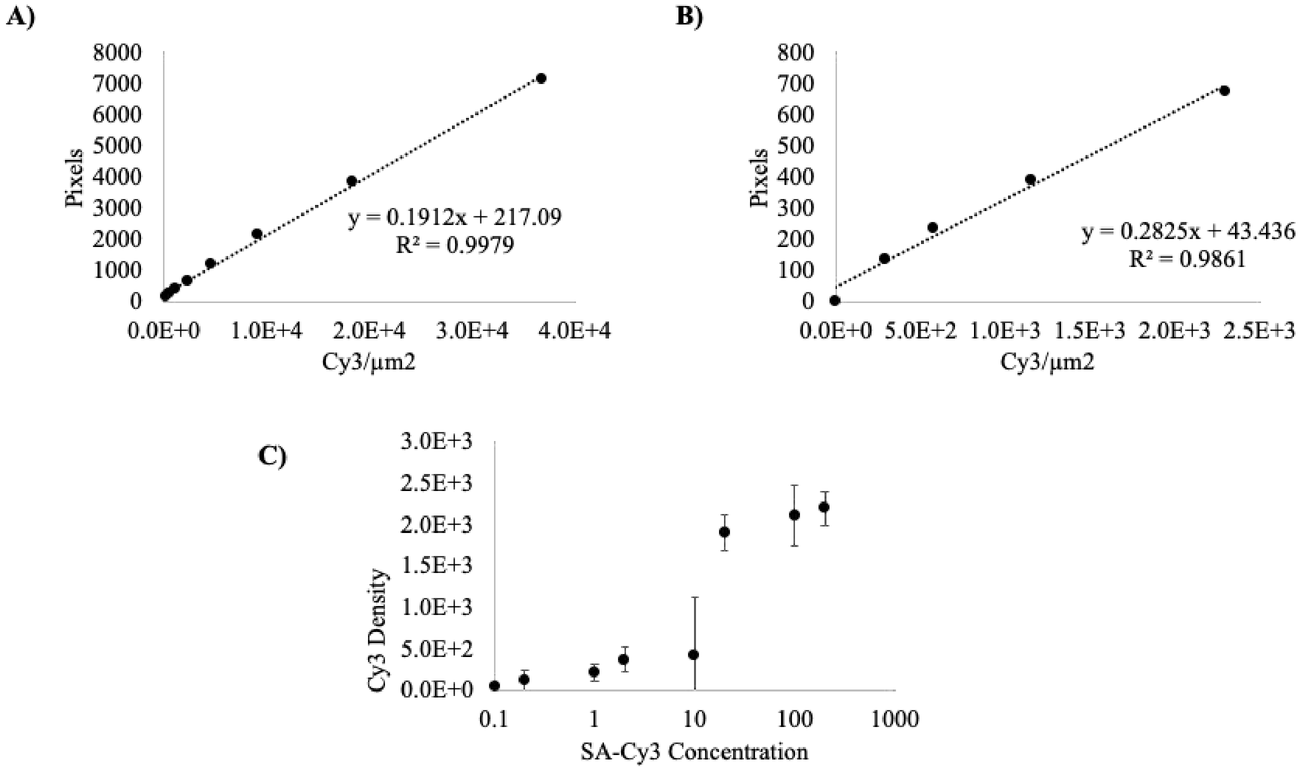

Figure S1. A) Higher Limit Array Scanner calibration curve for density of Cy3 per image pixels. **B)** Lower Limit Array Scanner calibration curve for density of Cy3 per image pixels. **C)** Density of Cy3 conjugated to a freshly prepared epoxy slide at varying concentrations of SA-Cy3.

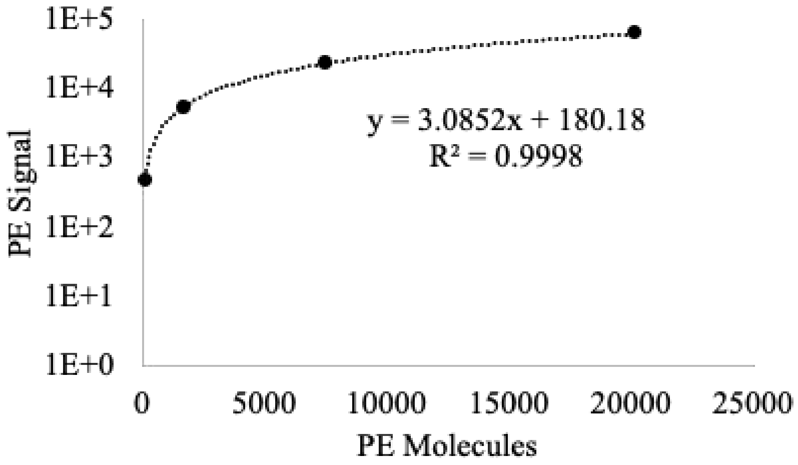

Figure S2. Calibration curve of PE signal measured by Flow Cytometry versus total PE molecules. Calibration determined using BD QuantiBRITE PE quantitation beads.

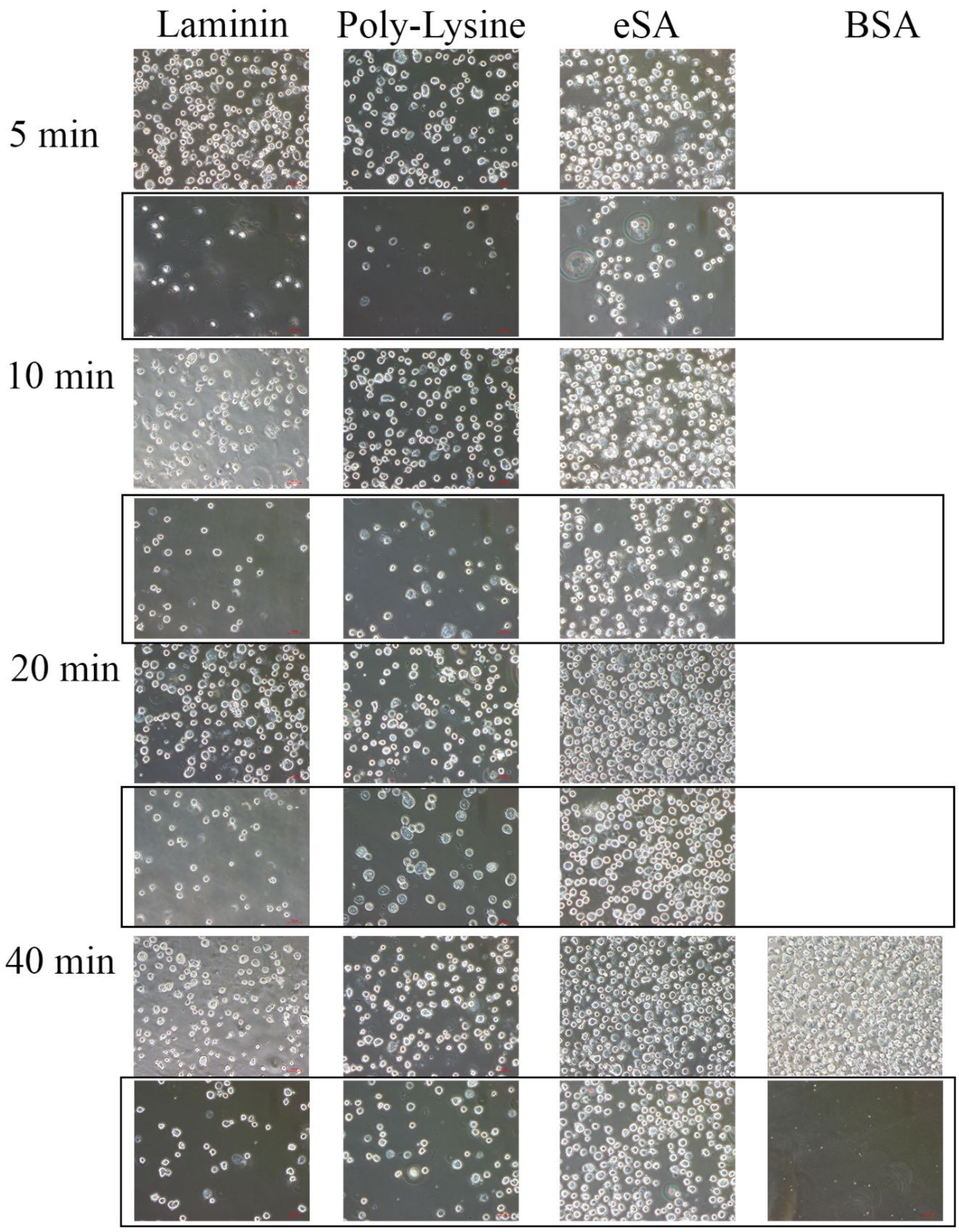

Figure S3. Enlarged images of H9C2 retention on various substrates following PBS rinsing (black box). Incubation times of 5, 10, 20 and 40 minutes (scale bar = 50 μm).

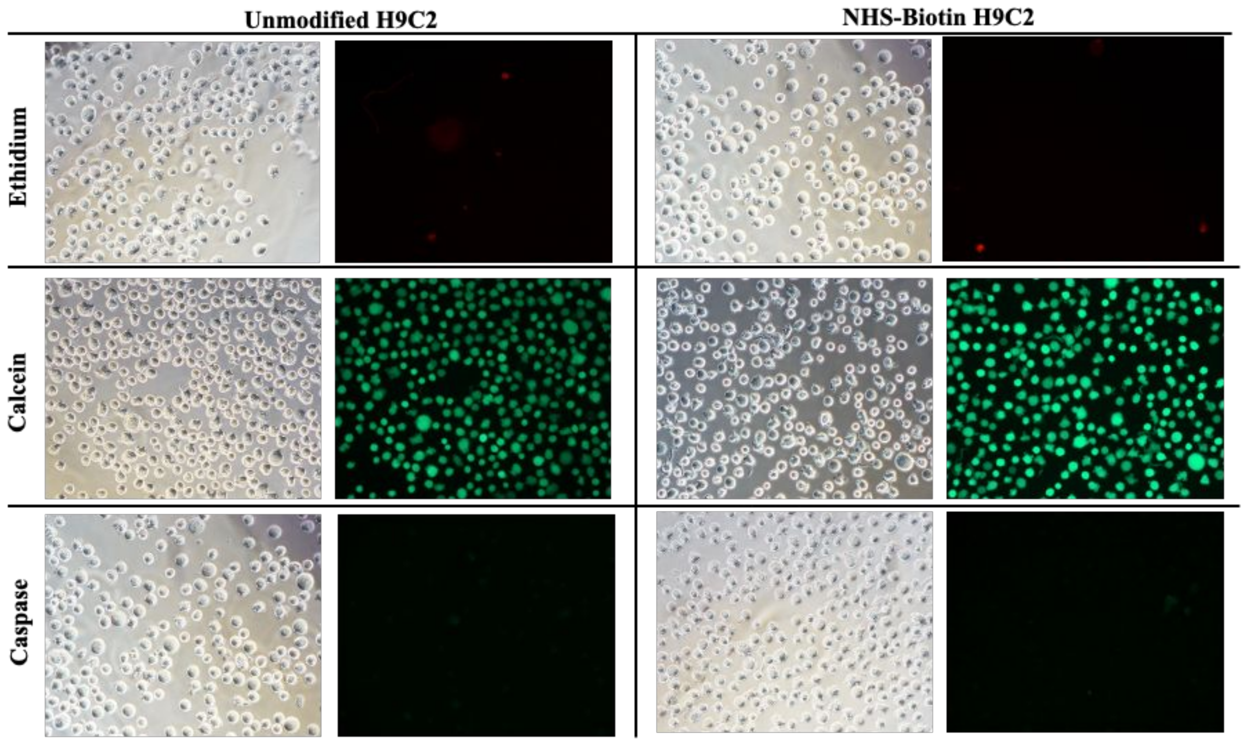

Figure S4. Microscopic images of bright field and fluorescent channel showing viability of unmodified H9C2 versus NHS-Biotin modified H9C2 through Calcein, ethidium and caspase assays.

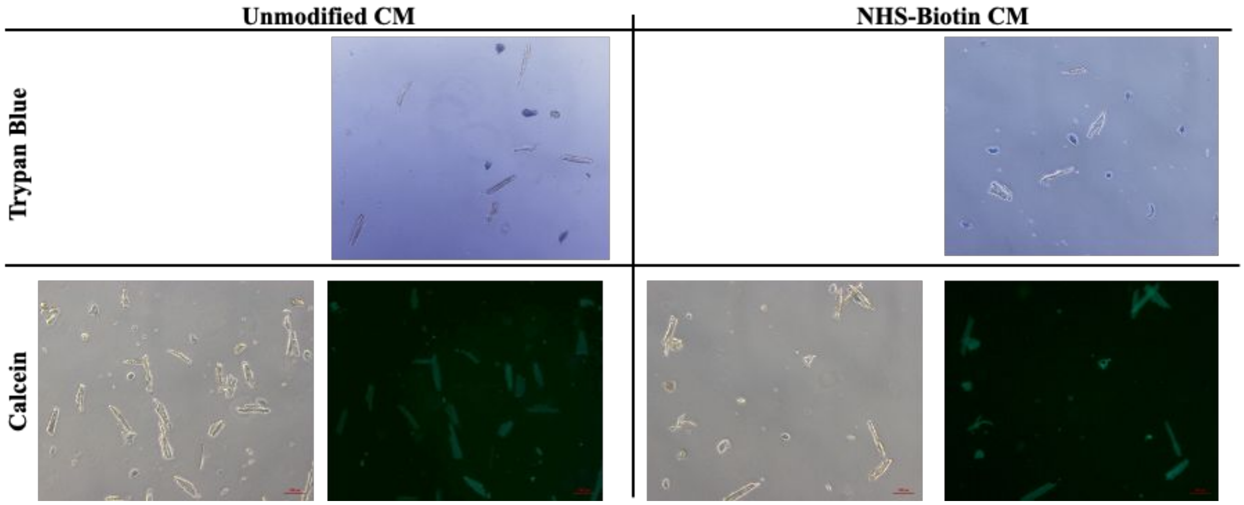

Figure S5. Microscopic images of bright field and fluorescent channel showing viability of unmodified CMs versus NHS-Biotin modified CMs through Calcein ant Trypan Blue.
